# Association of kidney disease measures with risk of renal function worsening in patients with type 1 diabetes

**DOI:** 10.1186/s12882-018-1136-6

**Published:** 2018-12-04

**Authors:** Antonio Mirijello, Francesca Viazzi, Paola Fioretto, Carlo Giorda, Antonio Ceriello, Giuspina T. Russo, Pietro Guida, Roberto Pontremoli, Salvatore De Cosmo, on behalf of the AMD ANNALS Study Group, Antonino Cimino, Danila Fava, Carlo Bruno Giorda, Illidio Meloncelli, Antonio Nicolucci, Fabio Pellegrini, Maria Chiara Rossi, Salvatore Turco, Giacomo Vespasiani, F. Pellegrini, G. Graziano, G. Lucisano, R. Memmo, E. Pellicciotta, V. Paciotti, M. Pupillo, G. Armentano, C. Giovannini, V. Armentano, M. Laudato, S. Turco, S. Acquati, A. V. Ciardullo, G. Laffi, G. Felace, C. Taboga, C. Tortul, G. Santantonio, C. Suraci, G. Ghisoni, M. Raffa, S. Genovese, C. A. Lovagnini-Scher, P. Rampini, A. Rocca, P. Ruggeri, E. Tortato, L. Cotti, M. R. Cristofaro, M. Tagliaferri, M. Comoglio, R. Fornengo, S. De Cosmo, F. M. Gentile, A. Gigante, F. Mastinu, A. Di Benedetto, P. Pata, A. Arcangeli, P. Orsini, P. Acler, G. De Blasi, G. Cicioni, S. Pocciati, A. Marangoni, A. Nogara, M. Lanero, M. G. Bertero, R. Damassino, C. Bergonzini, L. Schumtz, L. Seksich, A. Pipitone, M. Boaretto, I. Manfroi, L. Parmesan, B. Conte, F. Soccol, A. Pagano, E. Papini, R. Rinaldi, L. Petrucci, F. Graziano, M. Chianelli, S. Silvagni, M. Rosco, E. Ansaldi, F. Malvicino, M. Battezzati, P. Maresca, C. Palenzona, M. Boemi, R. A. Rabini, G. Brandoni, L. Lanari, C. Gatti, I. Testa, V. Cherubini, G. Doveri, L. Pecorelli, A. Ciccarelli, M. B. Gallardini, R. Courthoud, S. Sara Bredy, G. P. Ricciardi, G. Vitalone, D. Setti, P. Contrini, A. Corsi, V. Ghigliotti, G. Oddone, P. Ponzani, G. Valbonesi, V. Mazzini, P. Di Berardino, P. Colleluori, V. Montani, V. Trosini, M. Velussi, V. Paciotti, P. Alfidi, B. Verdecchia, L. Baliva, A. Di Pietro, G. Franchi, R. P. Luce, A. Marangoni, A. Pianta, M. Ferrari, S. Balzano, G. Beltranello, S. Dal Fabbro, C. N. Aricò, L. Cervo, R. Zanon, S. Rossa, M. Rosco, M. C. Di Pace, G. Laffi, A. Ciavarella, S. Giangiulio, M. Grimaldi, A. Mustacchio, G. Santacroce, B. Fattor, T. Monauni, M. Cristini, G. Orion, D. Crazzolara, F. Amor, J. E. Eisath, S. Lintner, S. Garavelli, T. Calari, P. Marini, O. Sandri, M. Scala, C. Stroppa, A. Trentin, S. Garavelli, T. Calari, P. Marini, R. Carlin, B. Carli, M. Sandonà, S. Garavelli, T. Calari, P. Marini, C. Zortea, L. Bonet, L. Pradel, S. Reato, M. Buschini, D. Bonfiglioli, D. Mones, F. Beldì, A. Morea, L. Bondesan, S. Perbellini, A. Cimino, U. Valentini, B. Agosti, R. Corsini, A. Girelli, E. Zarra, L. Rocca, G. De Blasi, M. Bergmann, I. Pradi, S. Unterkircher, M. Piok, M. Pichler, A. Trinchera, G. Palamà, P. Palma, L. Carboni, M. G. Murtas, T. Mudadu, M. P. Turco, M. Floris, A. Delogu, L. Farris, M. Songini, G. Piras, R. Seguro, R. Floris, G. Corona, M. Lai, E. Piras, P. P. Contini, S. Cocco, R. M. Pilosu, M. C. Sannia, F. Spanu, N. Busciantella Ricci, M. G. Cartechini, G. Agostinelli, C. Fiorelli, A. Nuzzi, C. Ballauri, C. B. Giorda, A. Lesina, F. Romeo, A. V. Ciardullo, G. Giudici, E. G. Maciejewska, A. Deroma, M. Paduano, L. Rossi, C. Vagnini, M. Dolci, M. Mori, F. Baccetti, G. Gregori, E. Straface, G. Pozzuoli, M. Laudato, M. Barone, G. B. Stasio, S. Tondini, F. Borgoni, J. Grosso, L. Rossi, C. Scarsellato, A. Sciulli, F. De Marco, L. Confortin, N. Marin, M. Lamonica, S. Gialdino, V. Borzì, C. Gatta, R. Rapisard, S. Strano, M. Calabrò, L. Puccio, M. Zolli, A. Coracina, V. Starnone, A. Del Buono, A. M. Terracciano, M. V. Monda, F. Castro, A. Guaglianone, V. Maccari, L. Corsi, G. Versari, M. R. Falivene, N. Boletto, S. Corsi, C. B. Giorda, L. Marafetti, E. Vitacolonna, F. Capani, L. Caputo, L. Di Nisio, F. Simonetti, A. Boscolo Bariga, A. Nogara, G. Ballarin, S. De Boni, S. Di Benedetto, A. M. Chiambretti, R. Fornengo, L. Di Vito, M. D. Pascuzzo, P. Urli, A. Rocca, P. Rumi, B. Balzarini, P. Galli, M. Castellan, A. Giannetti, C. Russotti, A. De Blasi, A. Perna, C. Campanelli, A. Ranchelli, D. Biccheri, G. Dadi, G. Santantonio, L. Massa, G. P. Baldi, F. Sciacca, E. Costanzo, M. Spada, G. Paolini, P. Ziller, F. Portolan, G. Pasolini, G. Ghilardi, P. Fiorina, M. L. Grata, L. Capretti, G. Speroni, L. Fugazza, C. Massafra, A. Lovagnini Scher, M. C. Cimicchi, C. Percudani, T. Risolo, P. Saccò, M. L. Grata, G. L. Gidoni Guarnieri, D. Piccolo, C. Bravin, E. De Noni, M. Scarpel, M. Marcon, F. Giacon, G. Panebianco, F. Tadiotto, V. Da Tos, M. D’Ambrosio, D. Pellizzola, M. A. Zampini, E. Frezzati, E. Mari, E. Raminelli, D. Gaiti, E. A. Bosi, G. Chierici, S. Pilla, M. Copelli, P. Zanichelli, L. Bertelli, P. Caretta, V. Vezzani, S. Bodecchi, A. Longobucco, P. Ruggeri, S. Di Lembo, E. Spotti, E. Carrai, A. Degli Innocenti, L. Manini, R. Persico, C. Rossi, G. Magro, G. Marelli, V. Vilei, M. Andrioli, L. Bellato, M. Fedeli, A. Merlini, G. Pinelli, G. Marin, M. L. Contin, A. Gallo, P. Parlato, W. Pecchielan, J. Jacovacci, G. Placentino, D. Richini, S. Molinari, R. Strazzeri, G. Panebianco, F. Tadiotto, V. Da Tos, M. D’Ambrosio, T. Fabbri, P. Di Bartolo, L. Cotti, G. Garrapa, F. D’Incau, P. Lagomanzini, P. Conte, F. Todesco, P. Foglini, E. Tortato, P. Pantanetti, C. Bedetta, R. Maricotti, F. Tomasi, M. Monesi, R. Graziani, F. Beretta, L. Penna, A. Guberti, D. Dazzi, M. Dolci, M. Mori, F. Baccetti, G. Gregori, S. Pocciati, E. Forte, A. Gasbarrone, T. Marrocco, R. Moschetta, F. Tuccinardi, F. De Meo, E. Forte, A. Coppola, P. Pirolozzi, E. Placitelli, R. Vallefuoco, C. Taboga, B. Catone, S. Ceschia, M. Urban, G. Ghisoni, F. Fabbri, M. Torresani, R. Crovetto, A. Corsi, M. Battistini, F. Fabbri, P. Carosia, G. L. Viviani, A. Durante, F. Pais, V. Lilliu, M. Rosco, C. Quieto, E. D’Ugo, M. Squadrone, T. Amenduni, M. M. Iovannisci, L. Della Penna, F. Potente, T. Delle Donne, C. Massa, M. A. Ulisse, S. De Berardinis, I. Guarnieri, S. Pace, M. Splendiani, R. Di Giuseppe, C. Tortul, B. Brunato, R. Assaloni, R. Muraro, R. Loro, S. Bucciol, M. Rosco, C. Lavacca, M. Rossi, G. Sabbatini, F. Quadri, L. Sambuco, C. Santacroce, E. A. Bosi, G. Chierici, S. Pilla, Gaiti, Copelli, Zanichelli, Bertelli, D. Paola Caretta, V. Vezzani, S. Bodecchi, C. Marino, A. Micheletti, A. Petrelli, A. Corda, L. Pisano, G. Guaita, C. Deias, G. Trevisan, I. Coletti, R. Iannarelli, M. Pupillo, A. De Luca, A. Minnucci, D. Antenucci, C. Di Florio, G. Angelicola, A. Bosco, R. Fresco, G. Di Marco, D. Ugolotti, T. Cadossi, M. Ferrari, M. Tagliaferri, P. Di Caro, M. Mazzocchetti, R. Buzzetti, G. Leto, C. Gnessi, L. Cipolloni, C. Foffi, C. Moretti, C. Venditti, A. Morea, L. Bondesan, S. Perbellini, R. Meniconi, S. Bertoli, S. Cosimi, G. Di Cianni, P. Orsini, A. Turco, A. Richini, S. Marconi, C. Sannino, P. Lemmi, S. Giuntoli, N. Manfrè, F. Giannini, A. di Carlo, I. Casadidio, P. Melandri, P. Di Bartolo, G. Maolo, B. Polenta, N. Piccinini, G. Pozzuoli, M. Laudato, M. Barone, G. B. Stasio, C. Vincenti, N. Pastore, P. Mega, E. Magurano, A. Cananiello, C. A. Francescutto, E. Brussa Toi, G. Gaspardo, L. Angeli, L. Ronchese, L. Sciangula, A. Ciucci, A. Contartese, E. Banfi, E. Castelli, P. Tatti, D. Bloise, P. Di Mauro, L. Masselli, A. Lo Presti, A. M. Scarpitta, F. Gambina, A. Venezia, R. Morea, G. Lagonigro, G. Copeta, V. Iannucci, V. Milano, M. Trupo, A. Lochmann, P. E. Marchetto, G. Incelli, G. De Paola, M. M. Steiger, M. A. Gamper, S. Breitenberger, M. Holzner, J. Frischmann, C. Lambiase, T. Di Vece, M. D’Aniello, M. Fezza, C. Giordano, F. Leo, G. Saitta, A. Di Benedetto, D. Cucinotta, G. Di Vieste, B. Pintaudi, P. Pata, T. Mancuso, N. Musacchio, A. Giancaterini, A. Lovagnini Scher, L. Pessina, G. Salis, F. Schivalocchi, G. Testori, P. A. Rampini, N. Cerutti, P. S. Morpugo, M. L. Cavaletto, G. Bonino, F. Morreale, G. Mariani, P. D. Ragonesi, P. Bollati, P. Colapinto, E. Bosi, L. Falqui, L. Bortolato, A. Cosma, P. Pistolato, B. Centenaro, A. Ceccato, G. Campobasso, F. M. Gentile, F. Zaurino, G. Mazzotta, M. Comoglio, R. Manti, C. B. Giorda, C. Tortul, R. Da Ros, S. Carlucci, L. Narduzzi, D. Bortolotto, L. D’Acunto, L. Stanic, B. Brunato, R. Assaloni, A. Volpi, A. Coracina, A. M. Cospite, V. Manicardi, M. Michelini, L. Finardi, F. Borghi, E. Manicardi, S. Lombardi, C. Tommasi, M. Iaccarino, S. Cozza, M. Binotto, F. Marini, I. Mecenero, S. Massignani, P. Stecco, E. Urbani, W. Massariol, R. Parolin, A. Gatti, M. Bonavita, E. Creso, R. Giannettino, M. Gobbo, S. Turco, C. Iovine, A. A. Turco, G. Riccardi, N. Iazzetta, C. Giannattasio, V. Armentano, O. Egione, S. Galdieri, A. Velotti, A. Azzolina, G. Annicelli, T. Sorrentino, I. Gaeta, A. Del Buono, L. Zenari, L. Bertolini, C. Sorgato, F. Grippaldi, M. Stroppiana, R. Popolizio, N. Carbone, S. Grasso, S. Abate, G. C. Gaggero, M. Strazzabosco, E. Brun, G. P. Carlesi, S. Garrone, A. Gigante, A. M. Cicalò, C. Clausi, R. Cau, A. Manconi, A. Carboni, M. F. Angius, A. A. Pinna, S. Caria, G. D. Filigheddu, G. Tonolo, I. Carta, S. Calebich, C. Burlotti, G. Saglietti, G. Placentino, A. Schellino, F. Mastinu, G. Madau, M. Cossu, F. Mulas, S. Zoccheddu, M. Balsanelli, M. Fetonti, A. Rotolo, P. Sambo, E. Secchi, M. A. Angotzi, S. Loddoni, I. Brundu, F. Careddu, A. Becciu, G. Gabriella Piras, F. Novara, F. Cipro, G. Torchio, P. Palumbo, A. Bianchi, G. Colucci, G. La Motta, A. Tiengo, A. Avogaro, D. Bruttomesso, C. Crepaldi, G. Fadini, G. Guarnieri, M. T. Lavagnini, A. Maran, M. Vedovato, V. de Kreutzenberg, D. Fedele, A. Lapolla, G. Sartore, G. Bax, C. Cardone, M. G. Dalfrà, M. Masin, R. Toniato, Francesco Piarulli, G. Mattina, M. A. Fulantelli, D. Gioia, M. Conti, G. Ridola, F. D’Agati, G. Grossi, F. De Berardinis, I. Zavaroni, A. Dei Cas, L. Franzini, E. Usberti, M. Antonimi, N. Anelli, R. Poli, V. Ridolfi, M. Michela, S. Haddoub, G. Prampolini, A. Muoio, M. C. Cimicchi, D. Ugolotti, D. Filippi, M. Ferrari, F. Bucci, S. M. Tardio, M. C. Calderini, M. G. Magotti, C. Quarantelli, M. A. Vernazza, A. Carolfi, R. Saracca, E. Picchio, P. Del Sindaco, A. Spalluto, L. Maggiulli, V. Torreggiani, S. Rastelletti, C. Ugolini, N. Pucci, S. Magi, S. Muratori, G. La Penna, A. Consoli, F. Galeone, A. V. Magiar, V. Gherardini, L. Moretti, M. Bientinesi, L. Landi, A. Bernardi, S. Del Prato, R. Miccoli, C. Bianchi, G. Penno, F. Venditti, R. Anichini, A. De Bellis, T. Bruschi, L. Butelli, M. Gioffredi, R. Gori, R. Picciafuochi, R. Malagoli, A. Bernini, R. Gelisio, M. Zanon, A. Del Bianco, A. Bamiston, M. Signorato, V. Mazzini, G. Citro, A. Arcangeli, M. Calabrese, L. Ianni, M. Lorenzetti, A. Marsocci, S. Guizzotti, G. Memoli, F. Cabasino, F. Farci, A. Atzori, A. Sanna, M. Ghiani, I. Siotto, M. Sedda, A. Manis, C. Loddo, I. Loddo, L. Pisano, P. Seguro, A. Cuomo, L. Orlando, G. B. Olanda, A. Pucci, M. Massenzo, P. Di Bartolo, C. Sardu, C. Giovannini, G. Perrone, F. Corazziere, I. La Puzza, P. F. Tripodi, S. Riggio, A. Giampaolo, D. Mannino, A. R. Aleandri, M. V. Guidi, B. Battisti, M. R. Faraglia, V. Lilli, S. Leotta, C. Suraci, N. Visalli, A. Gagliardi, L. Fontana, M. Altomare, S. Carletti, S. Abbruzzese, F. Chiaramonte, R. Giordano, M. Rossini, G. Migneco, D. Cappelloni, A. Urbani, F. Piergiovanni, D. Fava, A. Simonetta, F. Massimiani, R. Bulzomì, M. Giuliano, M. G. Pennafina, P. Di Perna, M. P. D’Accinni, D. Paolucci, A. D’Ubaldi, M. T. D’Angelo, G. Masaro, M. Pietrantoni, M. Fratini, R. La Rosa, M. Poggi, F. Piccirilli, R. Pisano, C. Saponara, I. Conforti, A. Penza, R. Scalpone, S. Lo Pinto, L. Iacovella, C. Caccamo, S. Sposito, C. Teodonio, G. Armentano, M. G. Restuccia, G. Mirto, R. Girardello, R. Gennaro, L. De Moliner, E. Bettini, A. Mattuzzi, K. Speese, F. Frisinghelli, S. Genovese, F. Locatelli, M. Nicoletti, N. Trojan, R. Centis, P. L Volsi, E. Levis, G. Zanette, G. Comba, L. Ballatore, A. Cattaneo, A. Aglialoro, R. Guido, M. Patrone, M. Zecchini, G. Vespasiani, I. Meloncelli, L. Clementi, M. Galetta, V. Marconi, P. Bordin, L. Perale, C. Vinci, M. Sira Zanon, L. Geretto, C. Toffolo, M. G. Furlan, G. Mazzanti, M. Vinci, R. Gelisio, V. Sica, M. Armeni, R. Derai, O. Ennas, S. Mamusa, M. A. Pisano, L. Carreras, S. De Cosmo, A. Rauseo, S. Cervone, A. Leggieri, M. Pontonio, R. Sturaro, M. Raffa, F. Quattrocchi, M. Molinaro, M. Trasatti, B. Ferretti, M. Rosco, G. Labarile, G. M. Baule, A. Gentilini, M. A. Spanu, A. Fancellu, P. Bianco, L. Lione, G. Massazza, G. Bocchio, E. Bosco, M. Monachesi, G. Carta, M. Boschetti, E. Ceresola, E. Venier, F. Calcaterra, F. Cataldi, M. Miola, S. Manfrini, A. Lai, B. Locci, D. Putzu, I. Tanganelli, M. Leonini, K. Egger, W. Marchiotto, L. Vincis, V. Orlandini, C. Pilloni, R. Farci, I. Pelligra, G. Renier, M. Mameli, A. Pala, E. Devigus, G. Felace, I. Fumagalli, C. Lalli, M. Leandri, M. Agliani, L. De Pascalis, F. Malci, A. De Ciocchis, M. B. Diodati, B. Macerola, S. Davì, A. Caccavale, L. Brocato, M. Pognant Gros, S. Borla, E. Lattanzi, C. Piersanti, A. Piersanti, I. Spinelli, L. Tuzzoli, V. Tulini, G. Quaranta, V. Iorio, M. Tirabovi, De Candia, G. Cicioni, M. G. Massarelli, S. Venturi, A. Travaglini, P. Draghi, P. Pomante, L. Richiardi, A. Clerico, A. Bruno, P. Cavallo Perin, E. Ghigo, M. Porta, P. Scuntero, R. Arcari, S. Bertaina, S. Bo, F. Broglio, G. Bruno, M. Degiovanni, P. Fornengo, G. Grassi, V. Inglese, M. Maccario, G. Maghenzani, S. Marena, V. Martina, P. Passera, G. Ruiu, M. Tagliabue, M. Zanone, M. Monge, G. M. Boffano, K. Macrì, P. Maio, A. Ozzello, E. Pergolizzi, D. Gaia, P. Gennari, G. Micali, E. Rossetto, C. Dalmazzo, M. Oreglia, T. Stefani, C. Dossena, P. Paglia, S. Bosoni, P. Acler, T. Romanelli, S. Inchiostro, M. Dauriz, C. A. Bossi, G. Meregalli, A. Balini, D. Berzi, B. Filippini, G. Crotto, A. Paccagnella, M. Orrasch, M. Sambataro, T. Citro, E. Kiwanuka, E. Bagolin, B. Almoto, A. Macchia, M. T. Branca, M. Filesi, R. Candido, E. Caroli, E. Manca, A. Petrucco, E. Tommasi, G. Jagodnik, B. Baskar, N. Daris, P. Dal Col, M. A. Pellegrini, L. Tonutti, G. Venturini, M. Andreani, F. Turchi, F. Fedrighelli, G. Martinelli, S. Sposito, R. Rongioletti, M. Candidi, M. Pais, E. Moro, F. Cervellino, R. Sinisi, A. Zampino, G. Saglietti, G. Placentino, A. Schellino, R. Mingardi, L. Lora, R. Reitano, C. Stocchiero, M. Strazzabosco, E. Brun, M. Simoncini, C. A. Mesturino, F. Zen, S. Di Pietro, C. Scoponi, L. Tilaro, S. Pelliccioni, R. Slongo, E. Vita, A. Garofalo, F. Vitale, B. Campanella, V. Mastrilli, A. Del Buono, T. Borrelli, A. D’Avino, A. Morea, A. Perbellini, L. Bondesan

**Affiliations:** 1 Department of Medical Sciences, Scientific Institute “Casa Sollievo della Sofferenza”, IRCCS Casa Sollievo della Sofferenza, v.le Cappuccini, 71013 San Giovanni Rotondo, FG Italy; 2 Department of Internal Medicine, University of Genoa and Policlinico San Martino, Genova, Italy; 3 Department of Medicine, University of Padova, Padova, Italy; 4 Diabetes and Metabolism Unit ASL Turin 5, Chieri, Italy; 5 Institut d’Investigacions Biomèdiques August Pii Sunyer (IDIBAPS) and Centro de Investigación Biomédicaen Red de Diabetes y Enfermedades Metabólicas Asociadas (CIBERDEM), Barcelona, Spain; 6 U.O. Diabetologia e Malattie Metaboliche, Multimedica IRCCS, Sesto San Giovanni, Milan, Italy; 7 Department of Clinical and Experimental Medicine, University of Messina, Messina, Italy; 8 Associazione Medici Diabetologi, Rome, Italy; 9 Department of Internal Medicine, University of Genoa and Policlinico San Martino, Genova, Italy

**Keywords:** GFR, Albuminuria, Diabetic kidney disease

## Abstract

**Background:**

Albuminuria has been classically considered a marker of kidney damage progression in diabetic patients and it is routinely assessed to monitor kidney function. However, the role of a mild GFR reduction on the development of stage ≥3 CKD has been less explored in type 1 diabetes mellitus (T1DM) patients. Aim of the present study was to evaluate the prognostic role of kidney disease measures, namely albuminuria and reduced GFR, on the development of stage ≥3 CKD in a large cohort of patients affected by T1DM.

**Methods:**

A total of 4284 patients affected by T1DM followed-up at 76 diabetes centers participating to the Italian Association of Clinical Diabetologists (Associazione Medici Diabetologi, AMD) initiative constitutes the study population. Urinary albumin excretion (ACR) and estimated GFR (eGFR) were retrieved and analyzed. The incidence of stage ≥3 CKD (eGFR < 60 mL/min/1.73 m2) or eGFR reduction > 30% from baseline was evaluated.

**Results:**

The mean estimated GFR was 98 ± 17 mL/min/1.73m^2^ and the proportion of patients with albuminuria was 15.3% (*n* = 654) at baseline. About 8% (*n* = 337) of patients developed one of the two renal endpoints during the 4-year follow-up period. Age, albuminuria (micro or macro) and baseline eGFR < 90 ml/min/m^2^ were independent risk factors for stage ≥3 CKD and renal function worsening. When compared to patients with eGFR > 90 ml/min/1.73m^2^ and normoalbuminuria, those with albuminuria at baseline had a 1.69 greater risk of reaching stage 3 CKD, while patients with mild eGFR reduction (i.e. eGFR between 90 and 60 mL/min/1.73 m2) show a 3.81 greater risk that rose to 8.24 for those patients with albuminuria and mild eGFR reduction at baseline.

**Conclusions:**

Albuminuria and eGFR reduction represent independent risk factors for incident stage ≥3 CKD in T1DM patients. The simultaneous occurrence of reduced eGFR and albuminuria have a synergistic effect on renal function worsening.

**Electronic supplementary material:**

The online version of this article (10.1186/s12882-018-1136-6) contains supplementary material, which is available to authorized users.

## Background

Diabetes represents the primary cause of end-stage renal disease (ESRD) in most of industrialized Countries [1]. It plays a synergistic role together with hypertension and ageing in the pathogenesis of renal disease [2]. The prevalence of diabetic nephropathy is constantly increasing [3, 4] accounting for a significant morbidity, mortality and reduction of Quality of Life (QoL) [5].

Diabetic kidney disease (DKD) is a serious complication of both forms of diabetes [4], involving about 25–75% of patients with type 1 diabetes (T1DM) [4, 6] and about 30–40% of those with type 2 diabetes (T2DM) [4]. Historically DKD has been defined as an increased urinary albumin excretion (i.e. microalbuminuria) that appears about 5 to 10 years after the onset of diabetes and, if untreated, progresses, in a period of 10–15 years, to macroalbuminuria (i.e. proteinuria) and to reduced glomerular filtration rate (GFR), and further on to ESRD [4]. Thus, the search and periodic evaluation of microalbuminuria for the diagnosis and follow-up of diabetic nephropathy has become part of routine clinical practice [7].

However, recent evidence suggests the increasing prevalence of a different pathway of DKD characterized by a decline of GFR (even) in the absence of micro and/or macroalbuminuria [6, 8–10].

Over the last decades, besides the identification of genetic predisposing factors [3, 11, 12], several environmental determinants [7] for the development and progression of kidney dysfunction in patients with T1DM have been described. Among these, hyperglycemia, dyslipidemia, increased blood pressure (BP) and smoking have been emphasized to bear a relevant unfavorable effect on renal function [6, 9, 13, 14].

In patients with T1DM, increased levels of albuminuria are not only a well-known manifestation of diabetic renal involvement, but also an established marker of renal damage progression. Longitudinal studies have demonstrated that GFR declines in parallel with the increase in urinary albumin excretion and Perkins and co-workers have shown a significant correlation between the rate of GFR decline with progressive stages of albuminuria [15]. In this context, the role of a mild, isolated GFR reduction on the development of stage ≥3 CKD has been less explored in T1DM patients.

Aim of the present study was to evaluate the independent prognostic role of kidney disease measures, namely albuminuria and reduced GFR, on the development of stage ≥3 CKD in a large cohort of patients affected by T1DM.

## Methods

### Study setting, patients and data sources

The study population is represented by patients affected by T1D followed-up at 155 Italian diabetes centers participating to the Italian Association of Clinical Diabetologists initiative (Associazione Medici Diabetologi, AMD). Electronic medical records collected between January 2004 and June 2008 were analyzed. Data set of patients ≥18 years old, with a follow-up within 48 ± 6 months, and no missing data concerning BP values, estimated GFR (eGFR), baseline evaluation of HbA1c, albuminuria classification, triglycerides, HDL and LDL were evaluated.

**Fig. 1:**
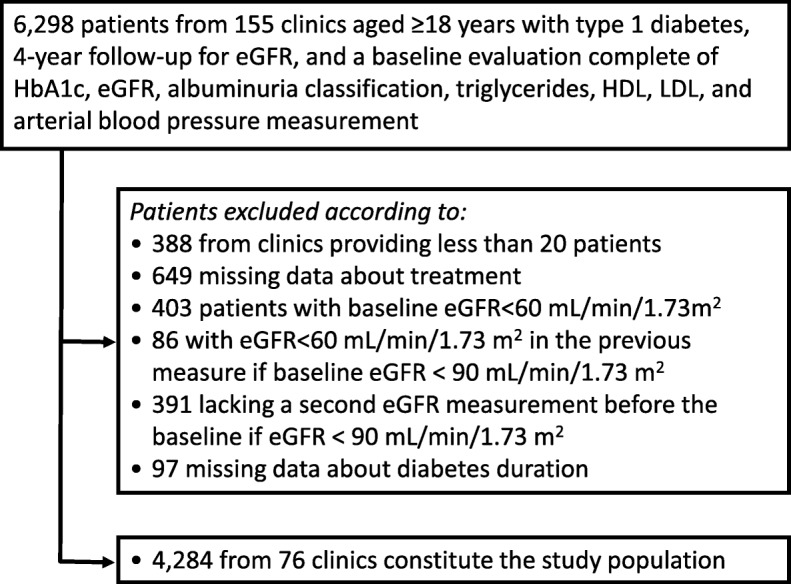
Study population: inclusion and exclusion criteria

Of 6298 patients identified, 2014 have been excluded for the reasons showed in Fig. 1.

The final study population is represented by 4284 patients affected by T1DM from 76 clinics homogeneously distributed throughout the Country.

### Methods and data collection

As previously reported [6, 9, 16–19], the Italian Association of Clinical Diabetologists (Associazione Medici Diabetologi, AMD) initiative and its clinical database were set up to recognize a set of indicators to be used for continuous quality improvement. The software for medical management of outpatients is the same among all centers. Patients’ data recorded are anonymized under a unique identification ID, encrypted to protect patients’ privacy. Clinical information collected from all centers can be anonymously extracted and centrally analyzed (AMD Data File) [16–19]. The automated system prevents the identification of individual patients. Thus, according to Italian law ethical committee approval and informed consent were not required. Results were internally revised and approved by AMD Annals Scientific Committee.

This initiative includes the measurement and monitoring of HbA1c, BP, low-density lipoprotein (LDL-c), total and high density lipoprotein cholesterol (HDL-c) and triglycerides, together with the evaluation of the use of specific classes of drugs (insulin, statins and two or more anti-hypertensive agents). Given the variability of normal ranges among centers, in order to allow comparisons, the estimated percentage change (measured value/upper normal limit) was multiplied by 6.0. The measurement of TG, performed by enzymatic-colorimetric or by ORTHO instrumentation had the same normal range. Even for HDL-C, normal range and decisional levels were the same.

Serum creatinine and urinary albumin excretion were used to assess kidney function. GFR was estimated by using a standardized serum creatinine assay and the Chronic Kidney Disease Epidemiology Collaboration formula [20]. Increased urinary albumin excretion was diagnosed as: i. microalbuminuria if urinary albumin concentration was > 30 and ≤ 300 mg/L, or if urinary albumin excretion rate was > 20 and ≤ 200 μg/min, or if urinary albumin-to-creatinine ratio (ACR) was > 2.5 mg/mmol in men and > 3.5 mg/mmol in women and ≤ 30 mg/mmol in both gender; ii. macroalbuminuria if urinary albumin concentration was > 300 mg/L, or if urinary albumin excretion rate was > 200 μg/min, or if ACR was > 30 mg/mmol in both gender. Albuminuria indicates patients with either micro or macroalbuminuria. In the present study, albuminuria determination was based on a single issue [21].

Physical examination and BP measurement were performed by a standardized protocol [6, 9, 16–19].

DKD was defined as diabetes with albuminuria or low eGFR (i.e. < 60 mL/min/1.73 m2) or both.

### Renal outcomes

The outcomes were: i. stage ≥3 CKD, i.e. an eGFR < 60 mL/min/1.73 m^2^; ii. eGFR reduction > 30% as compared to baseline; iii. The combination of either one of the two previous endpoints.

### Statistical analysis

Data are given as mean values ± standard deviation (SD); categorical variables are described as frequencies and percentages. Linear and logistic regression models were used to evaluate variables associated to, respectively, continuous and categorical data. To consider possible differences in data across diabetes clinics, mixed models were fitted with the enrolling centers as random effect. For each renal outcome were reported odds ratios (ORs) with their 95% confidence intervals (95% CIs). Multivariate models were fitted including a missing indicator variable (for BMI) and a complete-case analysis was also performed including only patients with all data available (models including serum uric acid and smoke status). The analyses were made using STATA software, version 14 (StataCorp, College Station, Texas). *P* values of < 0.05 were considered statistically significant.

## Results

Table 1 summarizes the main clinical features of the study population at baseline, overall and on the basis of the presence/absence of albuminuria. Table 2 summarizes the number and percentage of patients who reached one of the renal outcome at 4 year follow-up (Table 2).

**Table 1:** Baseline clinical characteristics of patients grouped by the presence of albuminuria

	All	Normoalbuminuria	Microalbuminuria	Macroalbuminuria	*p*
	*n* = 4284	*n* = 3630	*n* = 557	*n* = 97	
Male sex	2432 (56.8%)	2009 (55.3%)	363 (65.2%)	60 (61.9%)	< 0.001
Age (years)	45 ± 14	44 ± 14	47 ± 14	48 ± 12	0.006
Known duration of diabetes (years)	18 ± 12	17 ± 12	20 ± 12	25 ± 11	< 0.001
BMI (Kg/m^2^)	24.5 ± 3.6	24.4 ± 3.5	25.0 ± 3.7	25.4 ± 3.8	0.003
Serum creatinine (mg/dL)	0.84 ± 0.17	0.84 ± 0.16	0.86 ± 0.17	0.91 ± 0.21	< 0.001
eGFR (mL/min/1.73 m^2^)	98 ± 17	99 ± 17	96 ± 16	91 ± 18	< 0.001
Serum uric acid (mg/dL)	3.9 ± 1.4	3.8 ± 1.4	4.2 ± 1.2	4.6 ± 1.2	< 0.001
Serum uric acid in the top quintile	447 (18.3%)	361 (17.0%)	65 (24.5%)	21 (40.4%)	< 0.001
HbA1c (%)	7.8 ± 1.4	7.7 ± 1.4	8.0 ± 1.4	8.3 ± 1.4	< 0.001
HbA1c ≥ 7%	3071 (71.7%)	2555 (70.4%)	435 (78.1%)	81 (83.5%)	0.001
Total cholesterol (mg/dL)	190 ± 36	189 ± 35	188 ± 38	205 ± 42	< 0.001
Triglycerides (mg/dL)	87 ± 79	85 ± 81	97 ± 64	117 ± 70	< 0.001
Triglycerides ≥150 mg/dl	360 (8.4%)	275 (7.6%)	65 (11.7%)	20 (20.6%)	< 0.001
HDL (mg/dL)	62 ± 18	62 ± 18	60 ± 19	61 ± 17	0.089
HDL < 40 M <50F mg/dL	501 (11.7%)	408 (11.2%)	80 (14.4%)	13 (13.4%)	0.208
LDL (mg/dL)	110 ± 31	110 ± 31	109 ± 32	121 ± 35	0.001
LDL ≥100 mg/dL	2638 (61.6%)	2244 (61.8%)	324 (58.2%)	70 (72.2%)	0.012
Systolic BP (mmHg)	126 ± 17	125 ± 17	130 ± 19	137 ± 22	< 0.001
Diastolic BP (mmHg)	76 ± 9	75 ± 9	77 ± 9	80 ± 11	< 0.001
BP ≥ 140/85 mmHg	1310 (30.6%)	1039 (28.6%)	218 (39.1%)	53 (54.6%)	< 0.001
Non-proliferative retinopathy	925 (21.6%)	744 (20.5%)	149 (26.8%)	32 (33.0%)	0.018
Proliferative retinopathy	321 (7.5%)	232 (6.4%)	72 (12.9%)	17 (17.5%)	< 0.001
Smokers	602 (27.9%)	515 (27.3%)	72 (31.6%)	15 (38.5%)	0.119
Lipid-lowering treatment	861 (20.1%)	664 (18.3%)	162 (29.1%)	35 (36.1%)	< 0.001
Treatment with statins	822 (19.2%)	638 (17.6%)	153 (27.5%)	31 (32.0%)	< 0.001
Treatment with fibrates	19 (0.4%)	13 (0.4%)	4 (0.7%)	2 (2.1%)	0.045
Antihypertensive treatment	1102 (25.7%)	779 (21.5%)	249 (44.7%)	74 (76.3%)	< 0.001
Treatment with ACE-Is/ARBs	1001 (23.4%)	699 (19.3%)	231 (41.5%)	71 (73.2%)	< 0.001
Aspirin	425 (9.9%)	310 (8.5%)	93 (16.7%)	22 (22.7%)	< 0.001
Insulin pump	304 (7.1%)	269 (7.4%)	29 (5.2%)	6 (6.2%)	0.563

Mean ± SD or absolute frequency (percentage). *BMI* body mass index, *BP* blood pressure, *HbA1c* glycated haemoglobin, *HDL* high-density lipoprotein cholesterol, *LDL* low-density lipoprotein cholesterol, *eGFR* estimated glomerular filtration rate, *SBP* systolic blood pressure, *DBP* diastolic blood pressure, *ACE-Is* angiotensin converting enzyme-inhibitors, *ARBs* angiotensin II receptor antagonists. Patients’ baseline missing data: BMI in 176 (4.1%), serum uric acid in 1847 (43.1%), total cholesterol in 58 (1.4%), and smoking status in 2128 (49.7%). Serum uric acid the top gender-specific quintile: ≥4.0 mg/dL for females and ≥ 5.1 mg/dL for males. The *p* value refers to overall statistical significance of a mixed logistic regression model for categorical variables or mixed linear regression model for continuous variables with microalbuminuria and macroalbuminuria as covariate

**Table 2:** Four-year renal outcome of patients grouped by the presence of albuminuria

	All	Normoalbuminuria	Microalbuminuria	Macroalbuminuria	*p*
	*n* = 4284	*n* = 3630	*n* = 557	*n* = 97	
GFR < 60 mL/min/1.73 m^2^	238 (5.6%)	175 (4.8%)	43 (7.7%)	20 (20.6%)	< 0.001
GFR reduction > 30% than baseline	215 (5.0%)	154 (4.2%)	42 (7.5%)	19 (19.6%)	< 0.001
GFR < 60 or reduction > 30%	337 (7.9%)	255 (7.0%)	56 (10.1%)	26 (26.8%)	< 0.001

The mean age was 45 ± 14 years (56.8% male). Mean diabetes duration was 18 ± 12 years. The glyco-metabolic status of participants was rather unfair, being the mean values of HbA1c and LDL-c of 7.8 ± 1.4% and 110 ± 31 mg/dL, respectively. The average BP was 126 ± 17/76 ± 9 mmHg; about 30.6% of patients had BP > 140/85 mmHg at baseline. More than a quarter of patients were receiving antihypertensive treatment and 23.4% were taking an ACE-I or an ARB. The mean eGFR was 98 ± 17 mL/min/1.73 m2 and the proportion of patients with albuminuria was 15.3% (*n* = 654). Patients showing baseline albuminuria had a worse risk profile, such as longer duration of disease, higher BMI, serum uric acid, HbA1c, a more atherogenic lipid profile (i.e. higher triglycerides and LDL values). Moreover, they showed higher BP despite more antihypertensive and lipid lowering treatments.

Additional file 1: Table S1 reports the baseline clinical features of patients grouped on the basis of the achieved renal outcome within the study period. On average, patients who went on to develop low eGFR (i.e. < 60/ml/min/1.73m^2^ or eGFR reduction > 30%) showed a worse clinical profile. Older age, longer duration of diabetes and worse glycemic control were associated with the achievement of both renal outcomes while baseline eGFR was lower only among patients who developed stage ≥3 CKD. Patients who developed stage ≥3 CKD showed also significantly higher BP values at baseline, despite a higher prevalence of antihypertensive treatment, even with ACE-I or ARB.

**Fig. 2:**
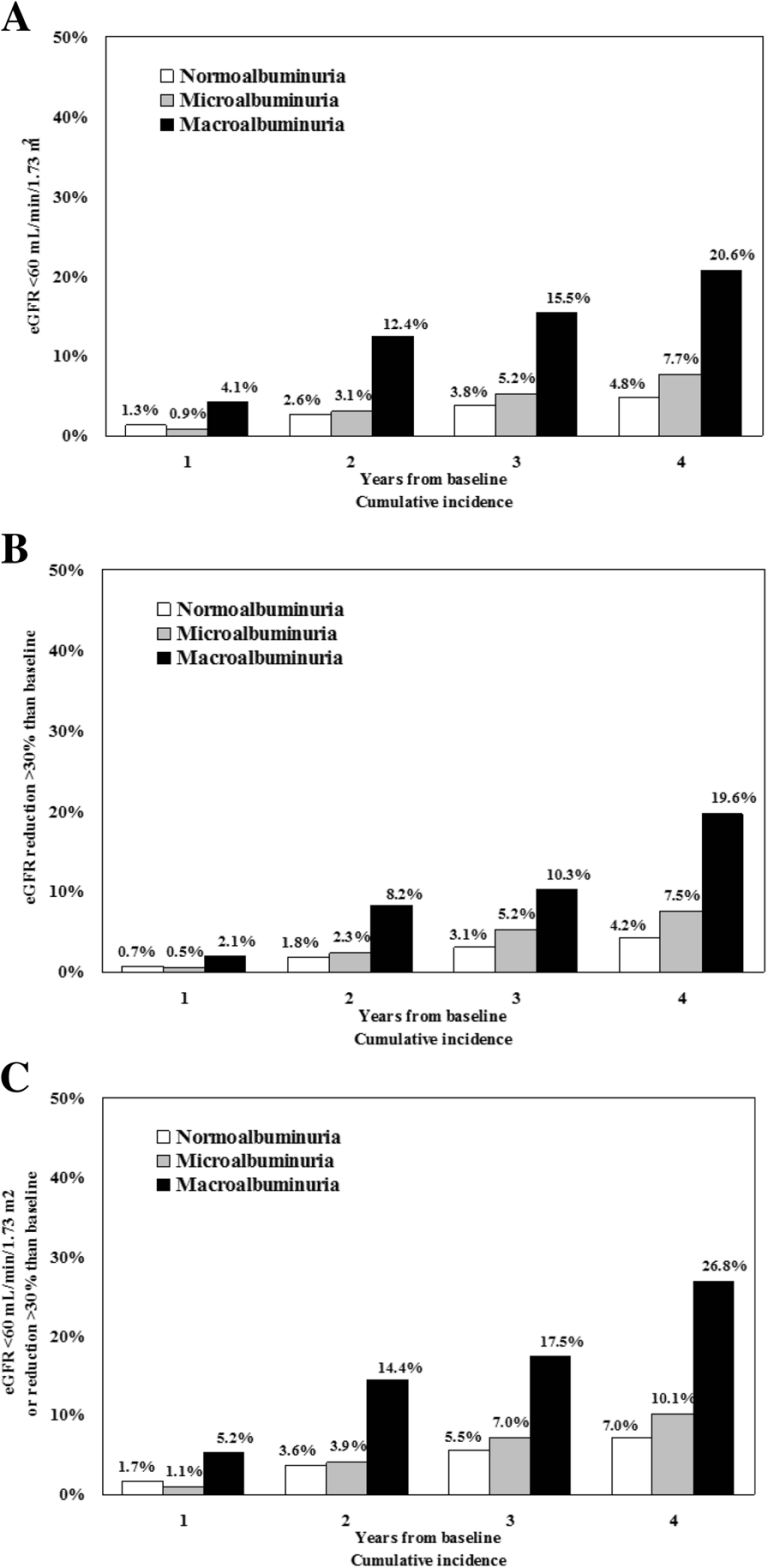
Year cumulative incidence of anyone of the two renal endpoints ((**a**) eGFR< 60 mL/min/1.73m^2^; (**b**) eGFR reduction > 30% from baseline; (**c**) both endpoints) during 4-year follow-up

The cumulative number of patients with a first occurrence of any of the two renal endpoints steadily increased during the study period (Fig. 2 a-c), with an incidence of 2.12% per year. Of the 4284 evaluated patients, 238 patients (5.6%) developed stage > 3 CKD within the 4-year follow-up period (Table 2 and Fig. 2a), while 215 patients (5%) showed a > 30% decrease in eGFR as compared to baseline (Table 2 and Fig. 2b) and 337 patients (7.9%) shower the combination of these two endpoints (Table 2 and Fig. 2c).

At multivariate analysis, age, albuminuria (micro and macro) and baseline eGFR < 90 ml/min/m2 remained independently and significantly associated to a higher risk of incident stage > 3 CKD and renal function worsening (Table 3). The duration of diabetes, BMI, baseline HbA1c and BP values did not significantly influence the outcome (Table 3). As for lipid profile, neither HDL nor LDL cholesterol levels significantly influence the outcome. On the contrary, a significant association with higher TG levels and eGFR reduction > 30% at 4-years was found (Table 3).

**Table 3:** Multivariate analysis of renal outcome within 4-year

	eGFR < 60 mL/min/1.73 m^2^		eGFR reduction > 30% than baseline		eGFR < 60 mL/min/1.73 m^2^ or reduction > 30%	
	Odds Ratio (95%CI)	*p*	Odds Ratio (95%CI)	*p*	Odds Ratio (95%CI)	*p*
Male sex	0.9 (0.65–1.24)	0.512	0.53 (0.39–0.71)	< 0.001	0.59 (0.46–0.76)	< 0.001
Age (by 10 years)	1.91 (1.65–2.22)	< 0.001	1.38 (1.21–1.58)	< 0.001	1.46 (1.30–1.63)	< 0.001
Duration of diabetes (by 5 years)	0.95 (0.89–1.01)	0.107	1.01 (0.94–1.08)	0.819	0.98 (0.93–1.04)	0.500
*BMI*						
27–30 Kg/m^2^	0.93 (0.61–1.43)	0.754	1.02 (0.68–1.53)	0.924	1.09 (0.77–1.53)	0.636
> 30 Kg/m^2^	1.16 (0.67–2.02)	0.599	0.92 (0.52–1.63)	0.770	1.15 (0.73–1.81)	0.543
*Albuminuria*						
Microalbuminuria	1.55 (1.00–2.40)	0.048	1.87 (1.27–2.76)	0.002	1.44 (1.01–2.06)	0.045
Macroalbuminuria	4.30 (2.19–8.46)	< 0.001	5.12 (2.81–9.35)	< 0.001	4.09 (2.33–7.16)	< 0.001
eGFR below 90 mL/min/1.73 m^2^ (by 5)	1.79 (1.64–1.96)	< 0.001	0.87 (0.78–0.97)	0.014	1.46 (1.36–1.57)	< 0.001
HbA1c ≥ 7%	1.08 (0.73–1.60)	0.695	1.17 (0.82–1.66)	0.386	1.13 (0.83–1.53)	0.437
Triglycerides ≥150 mg/dl	1.48 (0.88–2.50)	0.139	2.00 (1.28–3.12)	0.002	1.84 (1.24–2.74)	0.003
HDL < 40 M <50F mg/dL	0.83 (0.50–1.39)	0.480	1.09 (0.71–1.68)	0.695	1.04 (0.71–1.52)	0.835
LDL ≥100 mg/dL	1.08 (0.78–1.49)	0.651	0.83 (0.62–1.12)	0.229	0.93 (0.72–1.21)	0.596
BP ≥ 140/85 mmHg	1.12 (0.81–1.55)	0.503	1.35 (0.98–1.85)	0.064	1.24 (0.95–1.61)	0.118
Non-proliferative retinopathy	1.09 (0.75–1.58)	0.656	0.76 (0.52–1.10)	0.150	0.86 (0.63–1.17)	0.345
Proliferative retinopathy	1.42 (0.87–2.31)	0.162	1.19 (0.74–1.93)	0.477	1.18 (0.78–1.78)	0.443
Lipid-lowering treatment	0.87 (0.61–1.25)	0.464	0.85 (0.59–1.22)	0.370	0.87 (0.65–1.18)	0.373
Antihypertensive treatment	1.31 (0.90–1.90)	0.155	1.23 (0.86–1.77)	0.256	1.21 (0.90–1.65)	0.211
Aspirin	1.26 (0.83–1.91)	0.273	1.45 (0.95–2.23)	0.089	1.44 (1.01–2.05)	0.046
Insulin pump	1.76 (0.91–3.39)	0.091	0.97 (0.54–1.74)	0.909	1.21 (0.73–1.99)	0.461
*Separate models*						
Serum uric acid (mg/dL)	1.07 (0.96–1.20)	0.211	1.04 (0.94–1.16)	0.460	1.04 (0.95–1.15)	0.393
Serum uric acid in the top quintile	1.44 (0.90–2.31)	0.130	1.20 (0.75–1.89)	0.447	1.29 (0.88–1.90)	0.197
Smokers	0.67 (0.37–1.22)	0.190	1.14 (0.71–1.83)	0.599	0.84 (0.54–1.29)	0.423

Multivariate models were fitted including a missing indicator variable to indicate whether the BMI value is missing. Serum uric acid and smoking status evaluated in separate models that included patients with complete data

**Fig. 3:**
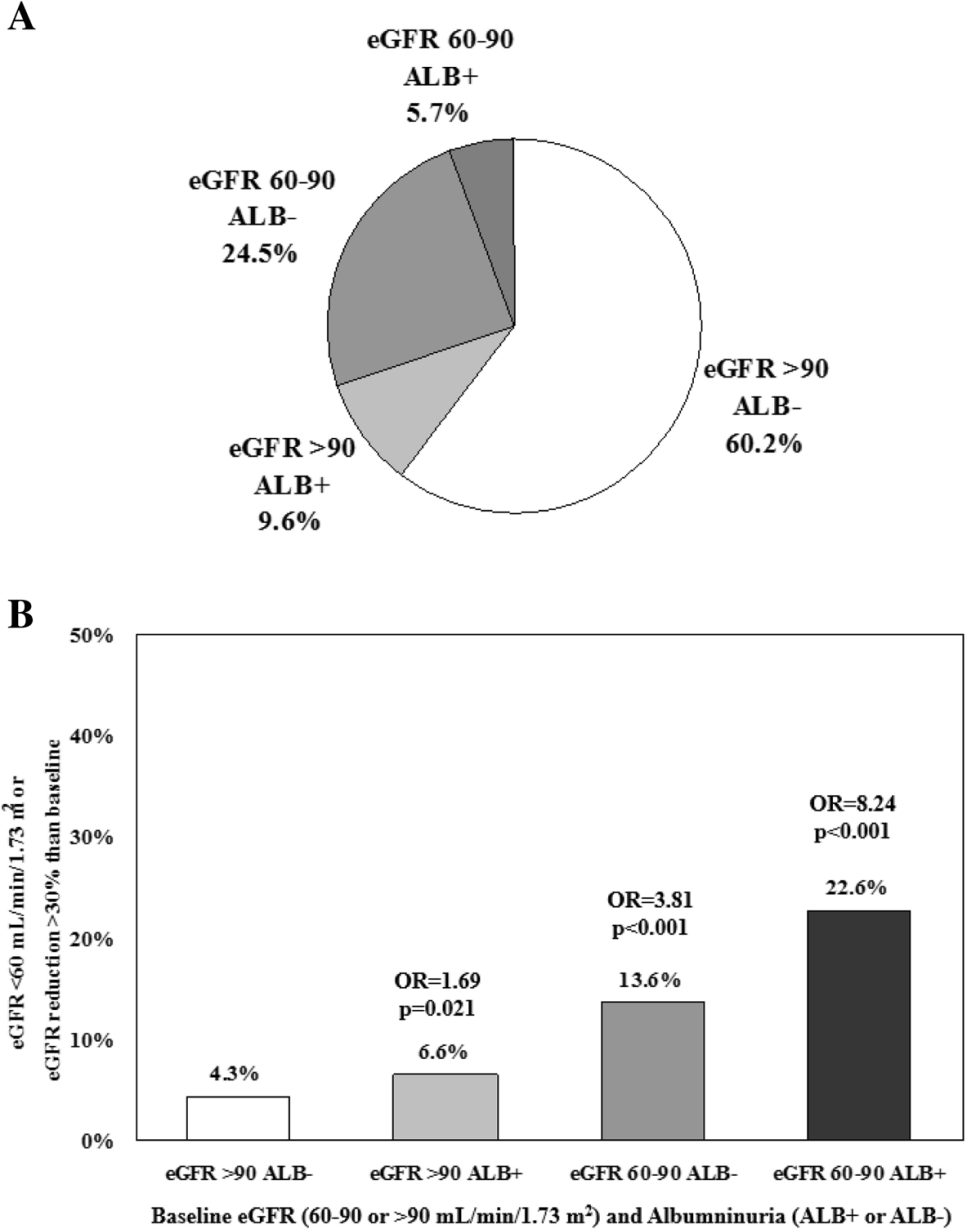
**a** Study patients stratified by baseline eGFR (> 90 mL/min/1.73m^2^/60-90 mL/min/1.73m^2^) and albuminuria (Alb+/Alb-); **b** Odd ratios of developing the composite renal endpoint over 4-year study period among different groups of patients

To better investigate the role of albuminuria and eGFR values on renal outcomes, patients were stratified based on the baseline eGFR (> 90 / 60–90) and albuminuria (Alb+/Alb-) (Fig. 3a, Additional file 2: Table S2). Among patients with eGFR values above or below 90 ml/min/1.73 m2 albuminuria was present in 15.9 and 23.2%, respectively (Additional file 2: Table S2). With respect to patients with eGFR > 90 ml/min/1.73m^2^ and normoalbuminuria (reference), the OR of developing the composite renal endpoint over 4-year study period was 1.69 (95%CI 1.08–2.64; *p* = 0.021) for patients with albuminuria and baseline eGFR values above 90 mL/min and rose progressively to 3.81 (95%CI 2.91–4.99; *p* < 0.001) in patients with normal albumin and eGFR values between 90 and 60 mL/min and then further up to 8.24 (95%CI 5.57–12.17; *p* < 0.001) when albuminuria was concomitant to mild GFR reduction (i.e. GFR between 90 and 60 mL/min) (Fig. 3b).

**Fig. 4:**
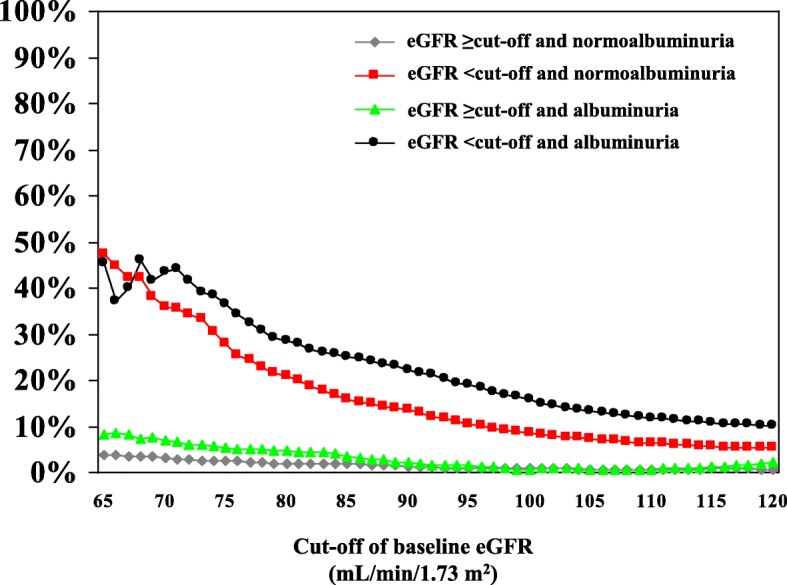
Estimated worsening rate of renal function at any given baseline eGFR

Figure 4 shows the estimated worsening rate of renal function at any given baseline eGFR. Albuminuria appears as an adjunctive risk factor for the progression to stage > 3 CKD over the entire study period.

**Fig. 5:**
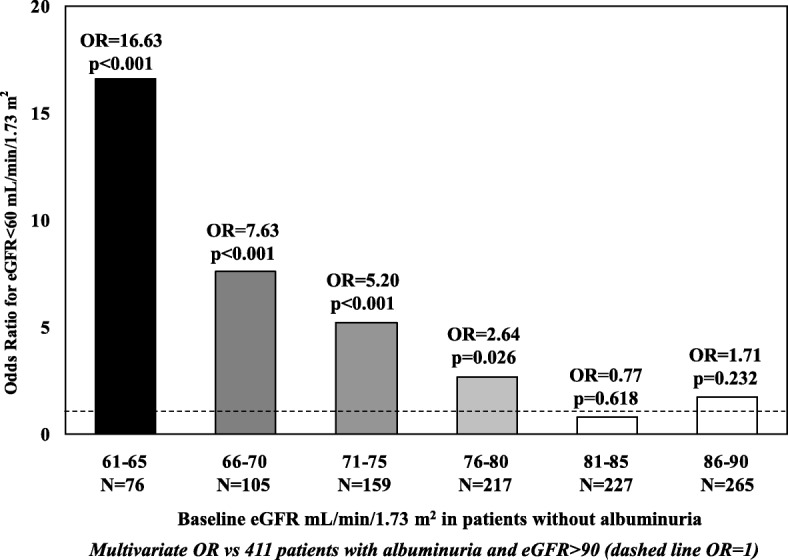
Multivariate OR for the incidence of stage ≥3 in patients with a given baseline eGFR and without albuminuria vs 411 patients with albuminuria and eGFR> 90 (dashed line OR = 1)

Multivariate OR for the incidence of stage > 3 CKD in patients with a given baseline eGFR and without albuminuria are showed in Fig. 5.

## Discussion

The present study shows that the cumulative incidence of stage ≥3 CKD observed over a 4-year follow-up in a large sample of patients affected by T1DM, representative of real-life Italian clinical practice, is 5.6%. In addition, both albuminuria and mild eGFR reduction independently predict a significant decline of renal function.

The observed rate of onset of stage ≥3 DKD is consistent with that previously reported in other cohorts of patients with similar characteristics [22–25].

Overall, patients who went on to develop stage > 3 CKD were older and had an unfavorable cardiovascular risk profile at baseline as compared to patients who did not develop renal dysfunction. The former, in fact, had a worse glycemic control, a more atherogenic lipid profile (i.e. lower HDL cholesterol and higher triglycerides), and higher levels of systolic and diastolic blood pressure despite the presence of a more aggressive antihypertensive treatment. Finally, micro or macroalbuminuria were more prevalent among patients who developed stage ≥3 CKD, who had also a lower baseline eGFR value.

In the present work, we have focused on the distinct role of albuminuria and mild eGFR reduction in promoting the onset of stage ≥3 CKD. In our dataset, albuminuria (i.e. micro or macroalbuminuria) appears to be a strong and independent predictor of kidney function loss. These data confirm and expand previous seminal observation by Viberti and co-workers [26] who first described the role of microalbuminuria as marker of early kidney damage and predictor of overt nephropathy in a small sample of patients with T1DM. Perkins and co-workers [15] several years later have shown among patients of the Joslin’s T1DM cohort that GFR decline starts early in the course of microalbuminuria. Recent results from DCCT by Molitch and co-workers [21] have further confirmed the role of albuminuria in accelerating GFR loss among T1DM patients although a not trivial proportion of patients (i.e. 24%), developed persistent eGFR < 60 ml/min/1.73 m2 in absence of albuminuria [22]. The pathophysiology and clinical significance of normoalbuminuric renal impairment is a current matter of debate.

In our study, the role of mild eGFR reduction in predicting the progression of kidney function decline appears to be remarkable. In fact, patients with eGFR < 90 ml/min/1.73 m2 show a constant and progressive increased risk to develop stage > 3 CKD as compared to patients with eGFR > 90 ml/min1.73 m2; the former presented 80% increased risk to develop eGFR < 60 ml/min for each 5 ml/min of eGFR decline. Although we’ve found that eGFR < 90 ml/min/1.73 m2 is an independent predictor for low eGFR (i.e. eGFR < 60 ml/min/1.73 m2), when we looked at the eGFR loss > 30% than baseline, it seemed to be protective as compared to patient with eGFR > 90 ml/min/1.73 m2 (Table 3). This finding is in line with data recently reported by Bjornstad and co-workers who studied a selected subset of 308 patients recruited from the Coronary Artery Calcification in Type 1 Diabetes (*N* = 210) and the Pittsburgh Epidemiology of Diabetes Complications studies (*N* = 98). The authors found that early renal function decline (defined as an annual loss of eGFR ≥3 ml/min/1.73 m2), which is an independent predictor of overt nephropathy, was associated with greater baseline eGFR levels. This finding was consistent across the two samples studied by Authors [27]. With this regard, no effect of hyperfiltration on microalbuminuria development in patients with T1DM was reported by Ficociello and coworkers at Joslin Diabetes Center [28]. On the contrary, Chiarelli and co-workers found that hyperfiltration predicted microalbuminuria over a 10-year follow-up in children and young adults with T1DM [29]. Finally, a meta-analysis aiming to unravel the role of hyperfiltration on diabetic nephropathy onset, concluded that individual with hyperfiltration are at increased risk of progression to diabetic nephropathy [30]. However, most of the knowledge on the mechanisms of hyperfiltration on the kidney come from animal models [31]. In those studies, hyperfiltration produces an increased glomerular pressure and a reduced flow, both mechanisms starting kidney damage [32, 33]. Although the underlying pathophysiological mechanism linking hyperfiltration and kidney function loss is not completely understood, the role of increased intraglomerular pressure associated to hyperfiltration could have a significant role [34]. Indirect information coming from renal protective effect of new antihyperglycemic drugs such as sodium-glucose cotransporter-2 seem to support this view [35].

The risk of developing stage > 3 CKD was independent of albuminuria, although it raised significantly to 8.2 times when albuminuria was present. This finding emphasizes the significant and independent role of mild eGFR reduction in inducing > 3 CKD, even though the presence of albuminuria entails an adjunctive risk.

In line with our finding, Caramori and co-workers [8] have shown that normoalbuminuric patients with long-standing type 1 diabetes (particularly females with retinopathy and/or hypertension), with reduced measured GFR defined as < 90 ml/min/ 1.73 m2, had worse diabetic glomerulopathy lesions than patients with similar duration of diabetes and normal or increased GFR, suggesting that careful GFR measurements may be indicated in normoalbuminuric patients, especially females with hypertension and retinopathy.

Our study has limitations and strengths. Among the first ones, the follow-up duration is relatively short, although the mean age of patients (i.e. 45 ± 14 years) and long duration of disease (i.e. 18 ± 12 years) allowed us to develop significant number of renal events. Second, laboratory measures, including serum creatinine, were not measured in a single, centralized laboratory. This could lead to some variability (i.e. GFR). Moreover, albuminuria has been gathered only as a categorical trait, possibly contributing to variability of the outcome measure. Furthermore, follow-up data were available for the majority, but not all patients. Thus, the generalization of our findings should be cautious, as mortality from competitive risk was not positively collected in the missing subgroup.

On the other hand, the large sample-size and the homogeneous clinical features of patients, as well as the representative geographical distribution of the recruiting centers, contribute to a good representation of Italian real life clinical conditions.

## Conclusions

Our data support the view that mild GFR reduction in patients with T1DM may be useful predictor of progression of GFR decline. Thus, microalbuminuria alone may not provide optimal identification of patients with T1DM at higher risk of renal impairment, measurements or estimation of GFR and identification of other risk factors will be surely useful.

## Additional files

Additional file 1: **Table S1.** Clinical characteristics of patients at baseline and at 4-year grouped by of basal GFR. (DOCX 20 kb)

Additional file 2: **Table S2.** Baseline clinical characteristics of patients grouped by eGFR and albuminuria. (DOCX 19 kb)

## Abbreviations

ACE-I: Angiotensin converting enzyme inhibitors
ACR: Albumin-to-creatinine ratio
AMD: Associazione Medici Diabetologi
ARB: Angiotensin receptor blocker
BMI: Body mass index
BP: Blood pressure
CKD: Chronic kidney disease
DCCT: Diabetes Control and Complications Trial
DKD: Diabetic kidney disease
ESRD: End-stage renal disease
GFR: Glomerular filtration rate
HbA1c: Glycated haemoglobin
HDL: High density lipoprotein
LDL: Low density lipoprotein
OR: Odds ratios
QoL: Quality of life
SD: Standard deviation
T1DM: Type 1 diabetes
T2DM: Type 2 diabetes
TG: Triglycerids
