# Supplementary material for: Association of kidney disease measures with risk of renal function worsening in patients with type 1 diabetes
Source: BMC Nephrol. 2018 Dec 4;19:347. doi: 10.1186/s12882-018-1136-6 (PMC6280443; doi:10.1186/s12882-018-1136-6)
Supplement: Supplementary file 1 — Table S1. Clinical characteristics of patients at baseline and at 4-year grouped by of basal GFR. (DOCX 20 kb) [file 12882_2018_1136_MOESM1_ESM.docx]

**Additional file 1: Table S1 Clinical characteristics of patients at baseline and at 4-year grouped by of basal GFR.**

|  | **eGFR<60 mL/min/1.73 m^2^** | | | **eGFR reduction >30% than baseline** | | | **eGFR<60 mL/min/1.73 m^2^ or reduction >30%** | | |
| --- | --- | --- | --- | --- | --- | --- | --- | --- | --- |
|  | **n=4046** | **n=238** | **p** | **n=4069** | **n=215** | **p** | **n=3947** | **n=337** |  |
| Male sex | 2315 (57.2%) | 117 (49.2%) | 0.681 | 2333 (57.3%) | 99 (46.0%) | 0.002 | 2282 (57.8%) | 150 (44.5%) | 0.005 |
| Age (years) | 44±13 | 60±13 | <0.001 | 44±14 | 51±16 | <0.001 | 44±13 | 55±15 | <0.001 |
| Known duration of diabetes (years) | 17±12 | 23±13 | 0.002 | 18±12 | 21±12 | <0.001 | 17±12 | 22±13 | 0.003 |
| BMI (Kg/m^2^) | 24.5±3.5 | 25.3±3.9 | 0.160 | 24.5±3.5 | 24.7±3.8 | 0.549 | 24.5±3.5 | 25.1±3.9 | 0.105 |
| Albuminuria | 591 (14.6%) | 63 (26.5%) | <0.001 | 593 (14.6%) | 61 (28.4%) | <0.001 | 572 (14.5%) | 82 (24.3%) | <0.001 |
| Microalbuminuria | 514 (12.7%) | 43 (18.1%) | 0.007 | 515 (12.7%) | 42 (19.5%) | <0.001 | 501 (12.7%) | 56 (16.6%) | 0.009 |
| Macroalbuminuria | 77 (1.9%) | 20 (8.4%) | <0.001 | 78 (1.9%) | 19 (8.8%) | <0.001 | 71 (1.8%) | 26 (7.7%) | <0.001 |
| Serum creatinine (mg/dL) | 0.84±0.16 | 0.94±0.18 | 0.024 | 0.84±0.16 | 0.79±0.19 | <0.001 | 0.84±0.16 | 0.87±0.21 | <0.001 |
| eGFR (mL/min/1.73 m^2^) | 99±16 | 78±14 | <0.001 | 98±17 | 98±18 | 0.799 | 99±16 | 87±20 | 0.359 |
| Serum uric acid (mg/dL) | 3.8±1.4 | 4.3±1.3 | 0.057 | 3.8±1.4 | 4.0±1.2 | 0.487 | 3.8±1.4 | 4.1±1.3 | 0.274 |
| Serum uric acid in the top quintile | 394 (17.2%) | 53 (36.6%) | 0.047 | 413 (17.9%) | 34 (25.0%) | 0.078 | 382 (17.1%) | 65 (32.0%) | 0.022 |
| HbA1c (%) | 7.7±1.4 | 8.1±1.4 | 0.006 | 7.7±1.4 | 8.0±1.5 | 0.005 | 7.7±1.4 | 8.0±1.4 | 0.004 |
| HbA1c≥7% | 2878 (71.1%) | 193 (81.1%) | 0.084 | 2903 (71.3%) | 168 (78.1%) | 0.048 | 2804 (71.0%) | 267 (79.2%) | 0.042 |
| Total cholesterol (mg/dL) | 189±36 | 196±36 | 0.068 | 189±35 | 194±40 | 0.032 | 189±36 | 195±37 | 0.038 |
| Triglycerides (mg/dL) | 86±80 | 97±51 | 0.240 | 86±80 | 97±60 | 0.154 | 86±80 | 97±57 | 0.093 |
| Triglycerides ≥150 mg/dl | 330 (8.2%) | 30 (12.6%) | 0.068 | 328 (8.1%) | 32 (14.9%) | 0.001 | 313 (7.9%) | 47 (13.9%) | 0.001 |
| HDL (mg/dL) | 62±18 | 65±19 | 0.177 | 62±18 | 64±20 | 0.093 | 62±18 | 64±19 | 0.182 |
| HDL <40M <50F mg/dL | 475 (11.7%) | 26 (10.9%) | 0.719 | 471 (11.6%) | 30 (14.0%) | 0.267 | 457 (11.6%) | 44 (13.1%) | 0.381 |
| LDL (mg/dL) | 110±31 | 111±30 | 0.547 | 110±31 | 110±33 | 0.838 | 110±31 | 111±31 | 0.701 |
| LDL ≥100 mg/dL | 2487 (61.5%) | 151 (63.4%) | 0.551 | 2513 (61.8%) | 125 (58.1%) | 0.521 | 2433 (61.6%) | 205 (60.8%) | 0.953 |
| Systolic BP (mmHg) | 125±17 | 137±20 | <0.001 | 126±17 | 132±22 | <0.001 | 125±17 | 134±21 | <0.001 |
| Diastolic BP (mmHg) | 75±9 | 77±10 | 0.053 | 76±9 | 75±10 | 0.717 | 76±9 | 77±10 | 0.314 |
| BP≥140/85 mmHg | 1186 (29.3%) | 124 (52.1%) | <0.001 | 1214 (29.8%) | 96 (44.7%) | <0.001 | 1150 (29.1%) | 160 (47.5%) | <0.001 |
| Non-proliferative retinopathy | 859 (21.2%) | 66 (27.7%) | 0.043 | 879 (21.6%) | 46 (21.4%) | 0.958 | 844 (21.4%) | 81 (24.0%) | 0.526 |
| Proliferative retinopathy | 286 (7.1%) | 35 (14.7%) | 0.001 | 294 (7.2%) | 27 (12.6%) | 0.004 | 278 (7.0%) | 43 (12.8%) | 0.006 |
| Smokers | 584 (28.6%) | 18 (16.1%) | 0.088 | 571 (27.8%) | 31 (29.8%) | 0.642 | 566 (28.4%) | 36 (21.7%) | 0.315 |
| Lipid-lowering treatment | 775 (19.2%) | 86 (36.1%) | 0.004 | 800 (19.7%) | 61 (28.4%) | 0.006 | 751 (19.0%) | 110 (32.6%) | 0.003 |
| Treatment with statins | 741 (18.3%) | 81 (34.0%) | 0.007 | 764 (18.8%) | 58 (27.0%) | 0.008 | 718 (18.2%) | 104 (30.9%) | 0.004 |
| Treatment with fibrates | 18 (0.4%) | 1 (0.4%) | 0.382 | 17 (0.4%) | 2 (0.9%) | 0.350 | 17 (0.4%) | 2 (0.6%) | 0.760 |
| Antihypertensive treatment | 969 (23.9%) | 133 (55.9%) | <0.001 | 1012 (24.9%) | 90 (41.9%) | <0.001 | 942 (23.9%) | 160 (47.5%) | <0.001 |
| Treatment with ACE-Is/ARBs | 880 (21.7%) | 121 (50.8%) | <0.001 | 916 (22.5%) | 85 (39.5%) | <0.001 | 854 (21.6%) | 147 (43.6%) | <0.001 |
| Aspirin | 363 (9.0%) | 62 (26.1%) | <0.001 | 381 (9.4%) | 44 (20.5%) | <0.001 | 348 (8.8%) | 77 (22.8%) | <0.001 |
| Insulin pump | 290 (7.2%) | 14 (5.9%) | 0.999 | 289 (7.1%) | 15 (7.0%) | 0.672 | 281 (7.1%) | 23 (6.8%) | 0.986 |

Mean±SD or absolute frequency (percentage).
