# Supplementary material for: Association of kidney disease measures with risk of renal function worsening in patients with type 1 diabetes
Source: BMC Nephrol. 2018 Dec 4;19:347. doi: 10.1186/s12882-018-1136-6 (PMC6280443; doi:10.1186/s12882-018-1136-6)
Supplement: Supplementary file 2 — Table S2. Baseline clinical characteristics of patients grouped by eGFR and albuminuria. (DOCX 19 kb) [file 12882_2018_1136_MOESM2_ESM.docx]

**Additional file 2: Table S2 Baseline clinical characteristics of patients grouped by eGFR and albuminuria.**

|  | **eGFR >90 mL/min/1.73 m2** | | **eGFR 60-90 mL/min/1.73 m2** | |  |
| --- | --- | --- | --- | --- | --- |
|  | **Albuminuria** | | **Albuminuria** | |  |
|  | **No** | **Yes** | **No** | **Yes** |  |
|  | **n=2581** | **n=411** | **n=1049** | **n=243** | **p** |
| Male sex | 1514 (58.7%) | 270 (65.7%) | 495 (47.2%) | 153 (63.0%) | <0.001 |
| Age (years) | 40±12 | 42±12 | 53±13 | 54±13 | <0.001 |
| Known duration of diabetes (years) | 16±11 | 18±11 | 21±13 | 25±13 | <0.001 |
| BMI (Kg/m^2^) | 24.3±3.5 | 24.7±3.7 | 24.7±3.6 | 25.7±3.7 | <0.001 |
| *Albuminuria* |  |  |  |  |  |
| Microalbuminuria | 0 (0.0%) | 360 (87.6%) | 0 (0.0%) | 197 (81.1%) |  |
| Macroalbuminuria | 0 (0.0%) | 51 (12.4%) | 0 (0.0%) | 46 (18.9%) |  |
| Serum creatinine (mg/dL) | 0.78±0.14 | 0.79±0.14 | 0.96±0.14 | 1.00±0.15 |  |
| eGFR (mL/min/1.73 m^2^) | 107±11 | 106±11 | 78±8 | 78±8 |  |
| Serum uric acid (mg/dL) | 3.7±1.4 | 4.1±1.2 | 4.0±1.3 | 4.5±1.3 | <0.001 |
| Serum uric acid in the top quintile | 204 (13.6%) | 43 (21.6%) | 157 (25.2%) | 43 (36.4%) | <0.001 |
| HbA1c (%) | 7.7±1.4 | 8.0±1.5 | 7.7±1.2 | 8.0±1.2 | <0.001 |
| HbA1c≥7% | 1778 (68.9%) | 321 (78.1%) | 777 (74.1%) | 195 (80.2%) | <0.001 |
| Total cholesterol (mg/dL) | 187±36 | 191±39 | 195±33 | 191±40 | <0.001 |
| Triglycerides (mg/dL) | 84±91 | 101±70 | 87±47 | 99±57 | <0.001 |
| Triglycerides ≥150 mg/dl | 186 (7.2%) | 57 (13.9%) | 89 (8.5%) | 28 (11.5%) | <0.001 |
| HDL (mg/dL) | 62±18 | 60±18 | 64±18 | 61±19 | <0.001 |
| HDL <40M <50F mg/dL | 286 (11.1%) | 53 (12.9%) | 122 (11.6%) | 40 (16.5%) | 0.193 |
| LDL (mg/dL) | 109±31 | 111±32 | 113±29 | 110±33 | 0.065 |
| LDL ≥100 mg/dL | 1557 (60.3%) | 250 (60.8%) | 687 (65.5%) | 144 (59.3%) | 0.058 |
| Systolic BP (mmHg) | 123±16 | 128±18 | 130±17 | 136±20 | <0.001 |
| Diastolic BP (mmHg) | 75±9 | 77±9 | 76±9 | 78±9 | <0.001 |
| BP≥140/85 mmHg | 646 (25.0%) | 151 (36.7%) | 393 (37.5%) | 120 (49.4%) | <0.001 |
| Non-proliferative retinopathy | 498 (19.3%) | 107 (26.0%) | 246 (23.5%) | 74 (30.5%) | <0.001 |
| Proliferative retinopathy | 135 (5.2%) | 47 (11.4%) | 97 (9.2%) | 42 (17.3%) | <0.001 |
| Smokers | 396 (29.5%) | 58 (33.5%) | 119 (21.8%) | 29 (30.9%) | 0.002 |
| Lipid-lowering treatment | 356 (13.8%) | 100 (24.3%) | 308 (29.4%) | 97 (39.9%) | <0.001 |
| Treatment with statins | 347 (13.4%) | 92 (22.4%) | 291 (27.7%) | 92 (37.9%) | <0.001 |
| Treatment with fibrates | 4 (0.2%) | 5 (1.2%) | 9 (0.9%) | 1 (0.4%) | 0.008 |
| Antihypertensive treatment | 434 (16.8%) | 158 (38.4%) | 345 (32.9%) | 165 (67.9%) | <0.001 |
| Treatment with ACE-Is/ARBs | 390 (15.1%) | 149 (36.3%) | 309 (29.5%) | 153 (63.0%) | <0.001 |
| Aspirin | 148 (5.7%) | 51 (12.4%) | 162 (15.4%) | 64 (26.3%) | <0.001 |
| Insulin pump | 203 (7.9%) | 20 (4.9%) | 66 (6.3%) | 15 (6.2%) | 0.483 |
|  |  |  |  |  |  |
| ***4-year renal outcome*** |  |  |  |  |  |
| GFR<60 mL/min/1.73 m^2^ | 32 (1.2%) | 9 (2.2%) | 143 (13.6%) | 54 (22.2%) | <0.001 |
| GFR reduction >30% than baseline | 112 (4.3%) | 27 (6.6%) | 42 (4.0%) | 34 (14.0%) | <0.001 |
| GFR<60 or reduction >30% | 112 (4.3%) | 27 (6.6%) | 143 (13.6%) | 55 (22.6%) | <0.001 |

Mean±SD or absolute frequency (percentage). The p value refers to overall statistical significance of a mixed logistic regression model for categorical variables or mixed linear regression model for continuous variables with eGFR and albuminuria groups as covariates.
